# Benefits of an Intensive Individual CO-OP Intervention in a Group Setting for Children with DCD

**DOI:** 10.1155/2022/8209128

**Published:** 2022-04-04

**Authors:** Hilde Krajenbrink, Jessica Lust, Jordi van Heeswijk, Pauline Aarts, Bert Steenbergen

**Affiliations:** ^1^Behavioural Science Institute (BSI), Radboud University, Nijmegen, Netherlands; ^2^Sint Maartenskliniek, Department of Pediatric Rehabilitation, Nijmegen, Netherlands; ^3^Centre for Disability and Development Research (CeDDR), School of Behavioural and Health Sciences, Australian Catholic University, Melbourne, VIC, Australia

## Abstract

**Purpose:**

The present study focused on the impact of an adapted Cognitive Orientation to daily Occupational Performance (CO-OP) five-day intervention program for children with developmental coordination disorder (DCD). Important adaptations were the new combination of individual CO-OP sessions and group activities, the short and intensive program that was followed by a training and coaching trajectory, and the use of video logs.

**Materials and Methods:**

Eighteen children with DCD (aged 8-16 years) participated in the five-day intervention during which they worked on three intervention goals. After the intervention, during an eight-week training and coaching trajectory for parents and children, children worked on a transfer goal. Assessment took place at four moments in time: two pretest measures, a posttest measure, and a 3-month follow-up measure. Primary outcome measures focused on changes in performance and satisfaction of self-chosen intervention and transfer goals. The secondary outcome measure explored changes in children's attitude, motivation, and confidence in relation to motor skill activities, social skills, and level of participation.

**Results:**

Significant improvements were found with regard to the performance and satisfaction of intervention goals. For the transfer goal, only parents reported significant improvements. Finally, parents indicated potential improvements with regard to the attitude, motivation, and confidence of their children, but not for their social skills or level of participation.

**Conclusion:**

The findings are promising with regard to the efficacy of this adapted CO-OP intervention for improving intervention goals, but less effective for transfer of learned skills to other goals after the intervention. Future research should focus on how postintervention parental coaching can be improved in order to increase generalization and transfer.

## 1. Introduction

Children with developmental coordination disorder (DCD) experience motor difficulties that have a wide and pervasive impact on their performance and participation in everyday life [1]. As a consequence, children with DCD have a higher risk for negative health and psychosocial outcomes such as obesity and low self-efficacy, compared with their typically developing peers [2, 3]. These negative outcomes necessitate effective intervention to enhance motor skill performance in children with DCD. Cognitive Orientation to daily Occupational Performance (CO-OP) is an intervention approach for children with DCD and focuses on guiding children to discover and apply cognitive strategies to solve motor problems and to facilitate skill acquisition [4]. The present study examines the impact of an adapted CO-OP intervention program for children with DCD. Important adaptations of the intervention under study are the combination of individual CO-OP sessions and group activities, the short duration of the program, the training and coaching trajectory for parents and children, and the video logs (vlogs) that children made throughout their training sessions.

Polatajko et al. [4] developed the original protocol of the CO-OP intervention for children with DCD. CO-OP is a task-specific and cognitive approach that facilitates skill acquisition by the application of a global cognitive problem-solving framework [5]. This framework is based on the verbal self-instruction strategy from Meichenbaum [6]. Therapists encourage and guide children to use the “*Goal-Plan-Do-Check*” strategy when performing challenging tasks. Children learn to formulate what task they want to complete (*Goal*), plan what strategies to use (*Plan*), try these strategies (*Do*), and evaluate their effectiveness (*Check*). Using this iterative process, children discover and evaluate domain specific strategies, in order to achieve their desired goals. These domain specific strategies are child, task, and situation specific.

Several studies demonstrated the effectiveness of the original individual CO-OP-based training sessions for individual children with DCD (e.g., [5]) as well as variations of CO-OP group interventions ([7], for a review). Benefits of group interventions are the opportunity to meet other children and families facing similar difficulties and challenges and to learn from peers [8]. However, if the group is heterogeneous with regard to the self-chosen goals, communication skills, and pace of progress, it may be difficult to create a collaborative atmosphere in which children support each other in developing domain-specific strategies. It has therefore been recommended to incorporate one-on-one sessions within a group format to mitigate this potential barrier [9]. This creates the opportunity to receive individual guidance, while participating in group activities with other children with DCD in a supportive environment [10].

The original CO-OP protocol consists of twelve weekly sessions of approximately one hour in length [4], but interventions have also been organized with weekly sessions over periods of, for example, 20 weeks [11], 10 weeks [12, 13], 9 weeks [14], and 7 weeks [15]. In addition, intensified approaches over a period of two weeks [10] and also four days [16] have been applied as well with similar positive outcomes with regard to goals that were trained during the intervention. In the latter study, the complete intervention was delivered over five days, but one of the days consisted of a day outing during which the goals of the children were not addressed.

Next to skill acquisition on trained goals, one of the basic objectives of the CO-OP approach is generalization and transfer of strategies to tasks and goals that are not directly trained during the intervention [4]. Several studies explored whether children demonstrated improvements on goals that were not addressed during CO-OP intervention sessions and showed mixed results; some children showed improvements on transfer tasks while others did not [16–18]. Martini and Savard [16] found no improvements on goals that were not directly trained after the four-day CO-OP group intervention that included a maximum of three individual CO-OP sessions. They suggest that this lack of transfer may be due to the little amount of individual guidance that children received by the therapists. Furthermore, they mention that it was difficult to practice tasks at home because of the high intensity of the intervention program. Next to homework activities, also the involvement of parents seems to play an important role in the generalization and transfer of learned skills. It was argued that differences in parent engagement in therapy sessions and parent support for ensuring opportunities to practice at home could explain differences in generalization and transfer between children [16–18].

Finally, an important factor in intervention programs, especially for children, is motivation and engagement [19]. Popular and entertaining modern techniques are increasingly applied in movement rehabilitation, such as the use of active video games [20]. One of the most important enabling principles of the CO-OP approach is to “make it fun.” This refers to the interaction style of the therapist as well as a range of techniques to make the process fun and engaging [4].

The present study focused on the impact of an adapted CO-OP intervention program “Vlog4Succes” [21]. In the intervention under study, the CO-OP protocol was intensified into a five-day program during which individual CO-OP sessions were applied in a group setting. In addition, parents were involved in the CO-OP sessions and in an individual eight-week training and coaching trajectory afterwards to promote generalization and transfer. Finally, the protocol was extended by the use of vlogs to increase children's motivation and engagement. The primary outcome measures focused on changes in performance and satisfaction of self-chosen goals. It was expected that performance and satisfaction of self-chosen goals would increase, both for goals trained during intervention and for goals trained during the training and coaching trajectory for parents and children afterwards. The secondary outcome measure consisted of a parent questionnaire to explore changes in children's attitude, motivation, and confidence in relation to motor skill activities, social skills, and level of participation.

## 2. Method

### 2.1. Participants

Twenty children took part in the intervention. All parents and children of 16 years of age gave written informed consent before participating in the study. In addition, children under the age of 16 gave assent for participation. Ethical approval was granted by the local ethics committee of the rehabilitation center. The study was considered not to be subject to the Medical Research Involving Human Subjects Act (WMO) by the Committee on Research Involving Human Subject of the region Arnhem-Nijmegen in The Netherlands (protocol number 2019-5745).

In order to be included in the data analysis of the present study, children had to complete the five-day program and comply with the DSM-V criteria for a DCD diagnosis [22]. Two children were excluded from the data analysis of the present study because the total Movement Assessment Battery for Children 2 (MABC-2; [23, 24]) score was >16^th^ percentile (*n* = 1) or because the child did not complete the intervention (*n* = 1). The final sample consisted of 13 boys and 5 girls with ages varying between 8y5m and 16y11m at the moment of the first pretest measure (*M* = 10y8m, SD = 2y8m). Of the 18 children, 17 children had a formal DCD diagnosis and one child had a developmental disorder of motor functions with a suspicion of DCD. The MABC-2 total score in percentiles of the included children varied between 0.10 and 16.00 (Median = 1.50, *M* = 4.21, SD = 5.21) and the Developmental Coordination Disorder Questionnaire (DCDQ'07, Dutch version; [25]) total score varied between 20.00 and 56.00 (Median = 40.00, *M* = 39.53, SD = 10.06). Detailed participant characteristics are presented in Table 1. One child was diagnosed with comorbid autism spectrum disorder. No other comorbid disorders were identified. Eight children also participated in the intervention a year before. Of this group, all but one child worked on self-chosen goals that were different from the goals that were trained in the year before (data was evaluated both with and without the 8 children that participated for the second time in the intervention; there were no differences in the pattern of results and it was therefore decided to include all children in the analyses).

### 2.2. Intervention

The intervention originated in a clinical setting and was organized for the second time. All therapists were previously trained in using the CO-OP approach and had a one-day refresher course in the week before the program started. A “beta version” of the present intervention was first pilot tested; after which refinements were made to the protocol as well as to the study procedures. This adapted intervention was used in the present study. The intervention adhered to the key principles of the CO-OP approach as described in Polatajko et al. [4] with some modifications that will be explained below.

**Figure 1:**
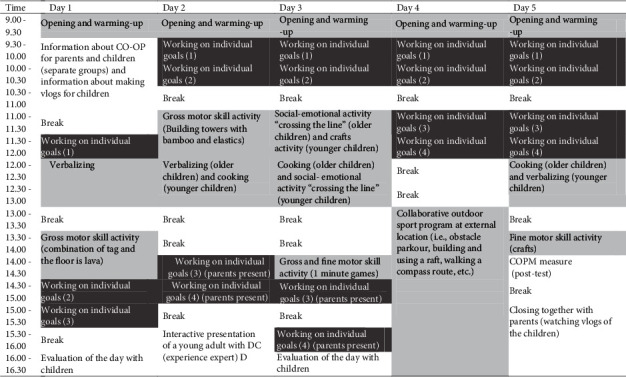
Time table of the intervention protocol with individual CO-OP sessions in dark grey, group activities in light grey, and other activities in white.

The intervention program (visually depicted in Figure 1) was conducted across five subsequent days, from 9:00 am to 4:30 pm. Before the intervention started, each child formulated four goals during a Canadian Occupational Performance Measure (COPM; [26]) interview together with a therapist and the parent(s). Three of the four goals were trained during the five-day intervention (i.e., “*intervention goals*”), while the fourth goal was trained during a training and coaching trajectory for parents and children after the intervention (i.e., “*transfer goal*”). This was determined in consultation with parents and children. Each day, children received three or four 30-minute sessions of individualized (child to therapist ratio 1 : 1) CO-OP intervention to work on the intervention goals. This resulted in a total of 9.5 hours of individual CO-OP therapy over the five-day intervention. For each goal, the child was coupled to a therapist based on therapists' expertise (e.g., a physiotherapist was assigned to a child whose goal was to run faster and an occupational therapist was assigned to a child whose goal was to tie shoelaces). Children and therapists remained working on the respective goals for the rest of the intervention.  Table 2 provides an overview of the types of goals that were trained and shows the number of children that worked on a certain type of goal.

At the start of the first day of intervention, the CO-OP approach was explained to the children. They received four cards illustrated with the four steps of the global problem-solving strategy, which provided visual support while practicing. During the individual CO-OP sessions, children worked on their intervention goals using the CO-OP techniques. In addition to the CO-OP key principles, at the end of each individual training session, children recorded at least one vlog in which they went through the global problem-solving strategy. Changes in domain-specific strategy use during a session were optionally recorded as well. This was done to increase children's motivation and engagement. During the five-day intervention, there were no structural homework assignments.

Next to the individual CO-OP training sessions, children were also involved in group activities. Group activities (child to therapist ratio varied from 1 : 3 to 1 : 5) were performed in two separate age-based groups or with the group as a whole. Most of these group activities (i.e., 11 hours in total) were focused on motor skills (i.e., sports, cooking, and crafts). During these group activities, therapists used the CO-OP approach as well. Although these activities were not specifically focused on children's self-chosen goals, for some children, these activities included their goals as well (e.g., in case a child's goal was to cut vegetables this goal was trained during the cooking activity). In addition, there were group activities focused on social-emotional skills (i.e., “crossing the line,” a game which led to personal conversations about topics such as bullying) and verbalization skills (i.e., practice to articulate goals and plans more clearly and precisely).

In line with the CO-OP key principles, parents were encouraged to be actively involved throughout the intervention program. Here, we use the terms “training” and “coaching.” Parents first received training in the use of the CO-OP approach which changed into coaching when parents understood the CO-OP principles [27]. At the beginning of the first day, parents attended a 1.5-hour presentation about the CO-OP approach. In addition, during the intervention, parents attended four individual sessions of their child. In these sessions, parents first observed the therapist using the CO-OP approach and were then encouraged to guide their children while coached by the therapist in doing so. On the final day, parents talked about their experiences with regard to the program and the CO-OP approach in two groups. During all meetings, parents were given the opportunity to ask questions and share ideas with the therapists or each other. After the intervention, there was a summer break of five weeks. Then, an eight-week training and coaching trajectory for parents and children started. During this trajectory, parents and children practiced the transfer goal at home using the CO-OP techniques and received 30 minutes of weekly online coaching by a therapist. Parents had to send a video of a practice session at home to the therapist each week. Based on this video and the questions that the parents and or children had, the therapist coached the parents and child by video calling with them during a practice session, phone calling with them outside of a practice session, or by responding to questions via the online chat. The main role of the therapist was to coach the family by providing guidance, but sometimes the therapist provided direct instructions to the family if this was needed. The therapist often provided a homework assignment for the family to complete in the upcoming week. The contact between the therapist and the family took place via a safe messaging app for healthcare professionals.

### 2.3. Outcome Measures

#### 2.3.1. COPM

The COPM [26] was used to measure changes in the self-perceived performance and satisfaction of self-chosen goals. Each goal is rated on two 10-point scales varying from 1 (not able to/not at all satisfied) to 10 (to do extremely well/extremely satisfied). Test-retest reliability of the COPM is good (ICC 0.84-0.92), and a minimal change of 2 points is considered clinically relevant [26]. Parents and children completed the COPM independently.

#### 2.3.2. PQRS-G

The Performance Quality Rating Scale Generic (PQRS-G; [5]) was used to measure occupational performance. The performance of the self-chosen goals was videotaped, and each goal was rated on a 10-point scale varying from 1 (cannot do the skill at all) to 10 (does the skill very well) by an independent rater. The independent rater was not involved in the intervention week and was blind to the time point of the videotaped goals. Test-retest reliability of the PQRS-G is moderate (ICC 0.71-0.77), and a minimal change of 2.13 is considered clinically relevant when scored by an occupational therapist [28]. Of all videotaped goals, a randomly chosen 10% was scored again by the first rater and 10% was scored by another rater. The intrarater reliability and the interrater reliability in our sample were moderate to good, with an average measure ICC of 0.79, 95% CI [0.58, 0.90], and 0.74, 95% CI [0.49, 0.87], respectively, calculated based on a mean rating, absolute agreement, two-way random effects model.

#### 2.3.3. Parent Questionnaire

A short parent questionnaire was used to explore changes in children's attitude, motivation, and confidence in relation to motor skill activities, social skills, and participation. The questionnaire consisted of five questions focused on children's attitude in relation to motor skill activities (i.e., “compared to before the intervention week, my child's attitude towards motor skill activities…”), children's motivation in relation to motor skill activities (i.e., “compared to before the intervention week, my child's motivation to undertake motor skill activities…”), children's confidence in relation to motor skill activities (i.e., “compared to before the intervention week, my child's self-confidence with regard to motor skill activities…”), children's social skills (i.e., “compared to before the intervention week, my child's social skills…”), and children's level of participation (i.e., “compared to before the intervention week, participating with peers…”). For each question, parents had to answer on a 7-point scale ranging from −3 (became much worse) to +3 (became much better) after which they were asked for a short comment to support their answer.

### 2.4. Procedure

**Figure 2:**
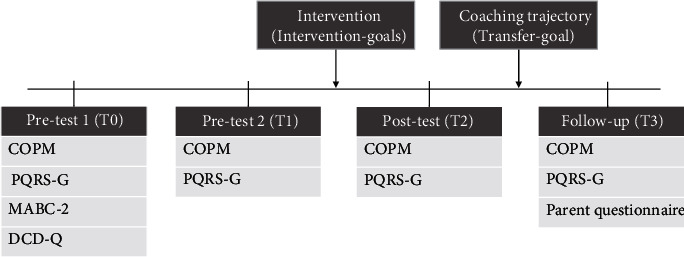
Overview of assessments over time. COPM: Canadian Occupational Performance Measure; PQRS-G: Performance Quality Rating Scale Generic; MABC-2: Movement Assessment Battery for Children 2; DCD-Q: Developmental Coordination Disorder Questionnaire.

The study consisted of a quasiexperimental one-group pretest-posttest design and took place in a clinical setting. Children were assessed at four time points (see Figure 2). There were two pretest measures before intervention started; pretest 1 (T0) took place at the rehabilitation center four weeks before the intervention started and pretest 2 (T1) took place at the home of the child in the week before the intervention started. The second pretest was included to establish a baseline to ensure changes in performance are not a result of maturation in the absence of a control group. The posttest (T2) was scheduled at the afternoon of the last day of intervention, and the follow-up measure (T3) took place at the rehabilitation center three months after the intervention. The COPM and PQRS-G were assessed at all four time points. Parents completed the short questionnaire that was focused on changes in child behavior at T3 only.

### 2.5. Data Analysis

The measures were completed by all children on T0, T1, and T2. Data on T3 were missing for one child. There were no missing items on the COPM scores of the intervention goals. For the COPM scores of the transfer goal, eight children had at least one missing data point on the child or parent performance or satisfaction ratings. With regard to the PQRS-G, all children had at least one intervention goal that was videotaped. For the PQRS-G scores of the intervention goals, there were two children with at least one missing data point. This was due to the fact that it was not always possible to videotape a goal at one of the locations (e.g., it was not possible to film rope climbing at the child's home). With regard to the PQRS-G scores of the transfer goal, there were twelve children with a goal that was videotaped. Of these twelve children, six children had at least one missing data point. There were no missing items on the parent questionnaire.

All analyses were completed using SPSS version 25 [29]. To examine changes in the COPM and PQRS-G scores of the intervention goals, children's scores were first averaged across their intervention goals, after which the Friedman test was used to examine changes in scores on each of the outcome measures (i.e., child and parent performance and satisfaction ratings). Also, for the COPM and PQRS-G scores of the transfer goal, the Friedman test was used to examine changes in scores across measurement time. Here, listwise deletion was used (i.e., if a child had missing data on one time point, the child was excluded from the analysis). A Bonferroni correction was used to correct for multiple testing, resulting in alpha level of 0.005. The Wilcoxon signed-rank test was used as a post hoc test. To optimize statistical power, pairwise deletion was used in the post hoc tests (i.e., if a child had a missing data point, the child was included for all other comparisons for which there was data available). For the post hoc analyses, the Bonferroni correction resulted in an alpha level of 0.0125. The effect sizes were calculated using the formula *r* = *Z*/√*N*. Effect sizes ≥ 0.10 are considered small, ≥0.30 are considered medium, and ≥0.50 are considered large [30]. Changes in the secondary outcome measure are presented in a descriptive manner.

## 3. Results

### 3.1. COPM

**Figure 3:**
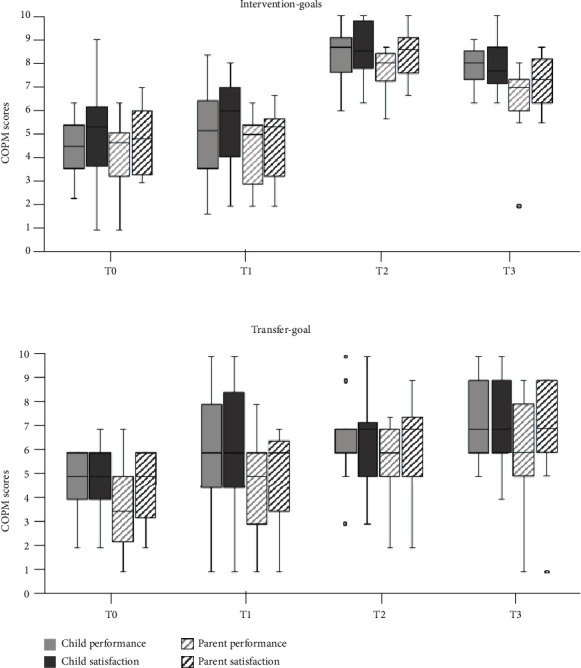
Changes in Canadian Occupational Performance Measure (COPM) scores over time for the intervention goals and transfer goal separately. The boxplots show the median, the first and third quartile, the range, and the outliers.

Results are visualized in Figure 3. With regard to the intervention goals, there was a significant change in scores over time for both child performance scores (*χ*^2^(3) = 39.94, p < 0.001) and child satisfaction scores (*χ*^2^(3) = 41.19, p < 0.001). Also, the parent performance scores (*χ*^2^(3) = 43.13, p < 0.001) and parent satisfaction scores (*χ*^2^(3) = 42.48, p < 0.001) changed significantly over time. Post hoc analyses showed that for all measures, there was a significant increase in scores after intervention with large effect sizes (Table 3). Considering individual child performance scores, all but one child reached clinically meaningful improvements on at least one goal of which eight children improved on all three goals. With regard to child satisfaction scores, all children reached clinically meaningful improvements on at least one goal, of which eight children improved on all three goals. Considering performance scores of parents, all but one child reached clinically meaningful improvements on at least one goal, of which twelve children improved on all three goals. Finally, for parent satisfaction scores, all children reached clinically meaningful improvements on at least one goal, of which thirteen children improved on all three goals. The scores at follow-up were still significantly higher compared to those at pretest. However, there was a small decrease in scores at follow-up compared to the posttest on all measures except for child performance ratings.

With regard to the transfer goal, there were significant changes over time in child performance scores (*χ*^2^(3) = 19.32, *p* < 0.001), but not for child satisfaction scores (*χ*^2^(3) = 11.19, *p* = 0.011). Post hoc analysis however revealed that child performance scores did not change significantly between the second pretest and posttest or follow-up. Individual child performance scores indicate that six out of the seventeen children reached clinically meaningful improvements on their transfer goal. With regard to child satisfaction scores, five out of the seventeen children reached clinically meaningful improvements. For the parent scores, both parent performance scores (*χ*^2^(3) = 12.98, *p* = 0.005) and parent satisfaction scores (*χ*^2^(3) = 13.45, *p* = 0.004) on the transfer goal changed significantly over time. Post hoc analysis showed that both parent performance and satisfaction scores increased at follow-up compared to the second pretest measure. Individual parent performance scores indicated that eight out of the fifteen children reached clinically meaningful improvements on their transfer goal. For parent satisfaction scores, nine out of the fifteen children reached clinically meaningful improvements.

### 3.2. PQRS-G

**Figure 4:**
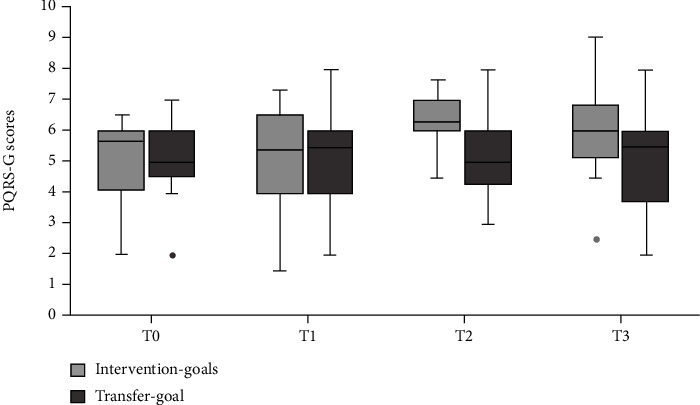
Changes in Performance Quality Rating Scale Generic (PQRS-G) scores over time for the intervention goals and transfer goal separately. The boxplots show the median, the first and third quartile, the range, and the outliers.

Results are visualized in Figure 4. With regard to the intervention goals, there was a significant change in scores over time (*χ*^2^(3) = 18.35, p < 0.001). Results of the post hoc analyses are represented in Table 4 and show that scores significantly improved after intervention. Out of the seventeen children, seven children improved with 3 points or more on at least one goal and four children improved with two points on at least one goal. With regard to the transfer goal, there were no significant changes in scores over time (*χ*^2^(3) = 0.29, p = 0.962). Here, out of the twelve children, four improved with two points on their transfer goal.

### 3.3. Parent Questionnaire

**Figure 5:**
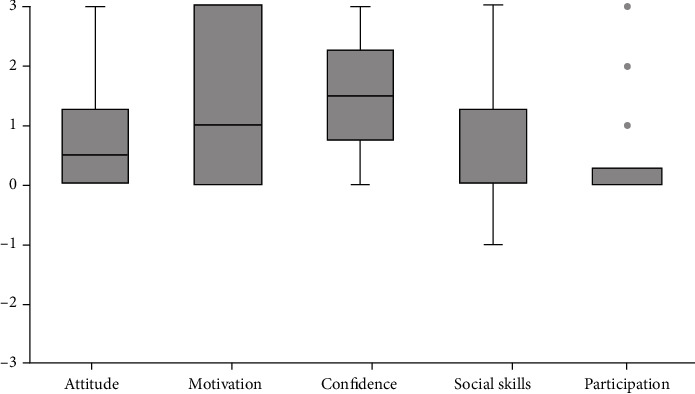
Median changes parents reported on a scale of -3 (became much worse) to +3 (became much better). The boxplots show the median, the first and third quartile, the range, and the outliers.

The median changes on a scale of -3 (became much worse) to +3 (became much better) that parents reported with regard to the attitude, motivation, and confidence towards motor activities, and social skills, and participation are presented in Figure 5. As can be seen, many parents reported improvements with regard to their child's attitude (11 out of 18), motivation (13 out of 18), and confidence (14 out of 18) in relation to motor skill activities. For social skills (5 out of 18) and participation (4 out of 18), less than half of the parents reported improvements.

## 4. Discussion

The aim of the present study was to examine the impact of an adapted CO-OP intervention for children with DCD. The intervention presented is unique in its combination of individual CO-OP sessions and group activities, the relative short duration, the involvement of parents, and the use of vlogs. Importantly and in line with our expectation, children and parents perceived improvements with regard to the performance and satisfaction of their self-chosen intervention goals. The improved performance was confirmed by independent therapist ratings. It was also examined whether children improved on tasks not directly addressed in the intervention, but during a coaching trajectory for parents afterwards. Here, the improvements were less convincing as only parents perceived significant improvements with regard to the performance and satisfaction of the self-chosen transfer goal. Finally, changes in areas other than motor skill performance were explored using a short parent questionnaire. Parents reported positive changes in their children's behavior with regard to the attitude, motivation, and confidence in relation to motor skill activities, but to a lesser extent for general social skills and participation. Below, we will discuss these results and provide suggestions for further research.

In line with and extending existing studies (e.g., [5, 13]), parents and children reported clinically meaningful and significant improvements with regard to self-chosen intervention goals after the intervention program. The size of these improvements in ratings is comparable with those in previous studies that used the COPM to evaluate the goals trained during a CO-OP intervention of two weeks [10]. Martini and Savard [16] did not find significant improvements in COPM scores after a CO-OP group intervention of four days. This may be due to the small number of individual CO-OP sessions that children received compared to the present intervention, but it could also be due to the small sample size in their study. Our results suggest that it is possible to achieve meaningful effects of CO-OP over a short period of five days. While these ratings are not an objective measure of motor performance quality, the results are promising and indicate that parents and children perceived increased motor performance and were satisfied with these improvements. In line with the perception of the parents and children, performance quality on self-chosen intervention goals also increased according to the ratings of an independent therapist as measured with the PQRS-G.

In addition to the performance and satisfaction ratings of goals that were directly trained during the intervention, it was also measured whether children improved on their transfer goal. This transfer goal was not addressed in the intervention, but during an individual eight-week coaching trajectory for parents and children afterwards. Contrary to the expectations, it was found that only parents perceived significant improvements with regard to the performance and satisfaction of the transfer goal. Although the performance and satisfaction scores of children improved as well over time, this change was not statistically significant. Finally, also for the quality performance ratings of the independent therapist (i.e., PQRS-G scores), there were no statistically significant improvements on the transfer goal. The individual variation in COPM scores on the transfer goal was much higher compared to that on the intervention goals. This is in line with previous studies that also found that while some children improved on their transfer goal, other children did not show any signs of improvement [16–18]. As has been previously suggested, this variation may be due to differences in the commitment and capacity of parents to support their children to apply the learned CO-OP strategies to other activities and goals outside the therapeutic setting. Contrary to these previous studies, however, parents in the present study received an individual training and coaching trajectory in order to support them in this role. Yet, although the overall involvement of parents in the present intervention was high, the therapists noticed that not all parents were equally engaged in the coaching trajectory. In addition, parents are no therapists and mentioned that they found it often difficult to guide their children according to the CO-OP approach and to find opportunities to practice at home. Furthermore, parents indicated children sometimes showed resistance to the help of parents. Future research should examine the amount of time that parents and children spend working on the transfer goal and their experiences in doing so more systematically. Another explanation for the limited improvements on the transfer goal may be that it was determined in consultation with children and parents which of the self-formulated goals would be the transfer goal. Possibly, the transfer goal was often the goal children and parents found least important or the goal they were least motivated for. In future research, the transfer goal should be decided upon (at random) by the therapist.

Next to the evaluation of self-chosen goals, a short parent questionnaire was used to explore changes in children's behavior with regard to the motivation, attitude, and confidence in relation to motor skill activities, social skills, and the level of participation. The majority of the parents indicated potential improvements with regard to the motivation, attitude, and confidence of their children in relation to motor skill activities. This is in line with previous research that also reported improvements in these areas after CO-OP intervention using in-depth interviews [31]. Contrary to the expectations, however, most parents did not report any improvements with regard to the social skills or the level of participation of their children. The majority of the parents reported no changes in their child's behavior. Of the parents that provided an explanation for their answer, some indicated that their child still experienced difficulties after the intervention. In these cases, it is likely that a five-day intervention was too short to have an impact on the social skills and level of participation of children, especially since this was not the main focus of the intervention. It should also be noted here that there were COVID-19 restrictions present in the period after the intervention which limited children's contact with peers and hampers interpretation of the results. Many parents also mentioned that there were no changes because the social skills and participation levels of their child were already high before the intervention started. This is in contrast to previous research in which it was found that children with DCD show lower levels of participation and tend to choose activities that are more socially isolated compared to peers [32]. This can possibly be explained by a selection bias as parents of children who find it hard to participate in a group may be less likely to choose a group intervention program. Importantly, it should be noted that this short parent questionnaire is not a standardized measure but was used as a tool to explore potential improvements in areas other than motor skill performance. In light of the present findings, it is warranted that future research should systematically examine whether the CO-OP intervention can indeed positively impact on children's attitude, motivation, and confidence in relation to motor skill activities, using standardized measures.

Finally, in the intervention under study, the CO-OP protocol was extended by the use of vlogs. These vlogs were introduced to potentially increase children's motivation to repeatedly verbalize the respective steps of the CO-OP approach. Although there was no systematic evaluation examining the effects of these vlogs, anecdotal observations support the claim that children found it both motivating and engaging to record the videos. In addition to being solely a tool to increase motivation, the vlogs allowed parents to view all of their child's CO-OP sessions (repeatedly) at home. Finally, the vlogs were optionally used by the children to evaluate their own performance and to check whether their plan had worked during the CO-OP sessions. In future studies, we advise to incorporate the vlogs more structurally in the individual CO-OP sessions to evaluate the effects more systematically and increase the involvement of parents.

The results of the study may have been impacted by two limitations. First, eight of the children in the study participated in the intervention for the second time and already received five days of CO-OP therapy one year before. These children may have benefitted from their previous participation resulting in higher improvements, but it could also have diminished potential improvements as these children already knew the CO-OP procedure. Still, we observed no difference in the pattern of results between these children and the children that participated for the first time in the intervention, deeming this limitation to be not too severe.

Second, not all measures were conducted by independent assessors. The involved therapists performed the COPM interviews with children and parents and recorded the videos that were used for the PQRS-G. To reduce the impact on the results, it was made sure that for the posttest and follow-up measure, the therapist performing the COPM interview did not work with the child on any of the child's goals during the individual CO-OP sessions.

Finally, the adapted CO-OP intervention under study has some benefits as well as some challenges with regard to its implementation in clinical practice. The main advantage of the new combination of individual CO-OP sessions and group activities was that it gave children the opportunity to receive individual guidance when working on personal goals, while at the same time they could interact with other children with DCD and learn from peers. Group heterogeneity with regard to age can sometimes present a challenge for the group activities (especially with outliers at the bottom or top), but this was counteracted by the amount of time spent on individual goals. Here, all children were able to work at their own level and pace of progress. The high intensity of the program contributed to the progress that the children achieved in a relatively short amount of time, but parents indicated that the long days could be demanding for the children. Due to these long days, it was decided to not include any homework assignments during the intervention week, but parents and children had the opportunity to practice together during some of the sessions as well as during the training and coaching trajectory that followed the intervention week. Taken collectively, we can conclude that this adapted CO-OP intervention protocol is promising and can be applied as a camp mode in summer/winter breaks in clinical practice.

## 5. Conclusions

Taken together, the present study demonstrates that the adapted CO-OP intervention under study was effective in improving self-chosen intervention goals as evaluated by children and parents as well as an independent therapist. The current intervention also showed some positive results on the motivation, attitude, and confidence of children in relation to motor skill activities. Improvements were limited with regard to the self-chosen transfer goal that was addressed after the five-day intervention during a training and coaching trajectory for parents and children. Future studies should focus on ways in which the generalization and transfer of learned strategies can be improved. Parents play an important role therein, and it should therefore be examined in what way parents' involvement can be increased and what parents need in order to help their children.

## Figures and Tables

**Table 1:** Detailed participant characteristics.

Child	Sex	Age range in years^∗^	MABC-2 total score in percentiles	DCD-Q total sum score
1	Male	8-10	0.10	22.00
2	Male	8-10	5.00	45.00
3	Male	8-10	2.00	56.00
4	Male	8-10	9.00	46.50
5	Male	8-10	9.00	33.00
6	Female	8-10	1.00	50.00
7	Male	8-10	0.10	46.00
8	Male	8-10	5.00	53.00
9	Male	8-10	0.50	39.00
10	Male	8-10	16.00	43.00
11	Female	8-10	0.50	39.00
12	Female	8-10	0.50	47.00
13	Male	8-10	5.00	29.00
14	Male	11-16	5.00	30.00
15	Male	11-16	0.50	34.00
16	Female	11-16	0.50	38.00
17	Female	11-16	0.10	20.00
18	Male	11-16	16.00	41.00

*Note*. MABC-2: Movement Assessment Battery for Children 2; DCD-Q: developmental coordination questionnaire; scores below 55 for 8- to 9-year-old children and below 57 for 10- to 15-year-old children are an indication for DCD. ^∗^Age range instead of exact age to protect the anonymity of the participants.

**Table 2:** Overview of the types of intervention goals and transfer goals that were trained and the number of children that worked on a certain type of goal.

Category	Types of goals	Example of a goal	
Gross motor skill activities	Cycling/skating	To sign with one hand while cycling	11
	Ball activities	To improve throwing a ball with one hand	7
	Climbing	To climb a rope	5
	Running	To run faster	3
	Jumping	To jump from a high obstacle	2
	Salto gymnastics	To perform a salto while landing on feet	2
	Other	To improve the performance of karate moves	5
Activities of daily living	Getting dressed	To tie shoelaces	6
	Personal hygiene	To brush own hair	5
	Food related	To improve slicing cheese	5
Organizational tasks	Planning day structure/agenda	To improve homework planning in agenda	7
	Working independently	To start with a task independently after an explanation	2
	Tidying and organizing	To improve tidying up clothes	2
	Other	To plan using the public transport	2
Fine motor skill activities	Writing/drawing	To write numbers in small boxes	4
	Arts and crafts	To cut more neatly	2
	Building Lego	To build with Lego following a booklet	2

**Table 3:** Post hoc analyses for the COPM scores of the intervention goals and transfer goal.

			*Z*	*p*	*r*
Intervention goals	Child performance	T0 vs. T1	-2.24	0.025	-0.37
		T1 vs. T2	-3.62	<0.001	-0.60
		T1 vs. T3	-3.51	<0.001	-0.60
		T2 vs. T3	-2.35	0.019	-0.40
	Child satisfaction	T0 vs. T1	-1.53	0.126	-0.26
		T1 vs. T2	-3.73	<0.001	-0.62
		T1 vs. T3	-3.53	**<0.001**	-0.61
		T2 vs. T3	-3.13	**0.002**	-0.54
	Parent performance	T0 vs. T1	-1.13	0.183	-0.22
		T1 vs. T2	-3.73	**<0.001**	-0.62
		T1 vs. T3	-3.49	**<0.001**	-0.60
		T2 vs. T3	-3.02	**0.003**	-0.52
	Parent satisfaction	T0 vs. T1	-0.16	0.874	-0.03
		T1 vs. T2	-3.73	**<0.001**	-0.62
		T1 vs. T3	-3.63	**<0.001**	-0.62
		T2 vs. T3	-2.54	**0.011**	-0.43
Transfer- goal	Child performance	T0 vs. T1	-1.00	0.319	-0.20
		T1 vs. T2	-0.99	0.322	-0.17
		T1 vs. T3	-1.71	0.087	-0.30
		T2 vs. T3	-1.75	0.080	-0.30
	Child satisfaction	T0 vs. T1	-1.39	0.165	-0.28
		T1 vs. T2	-1.03	0.303	-0.18
		T1 vs. T3	-1.29	0.196	-0.23
		T2 vs. T3	-1.07	0.286	-0.18
	Parent performance	T0 vs. T1	-2.33	0.020	-0.47
		T1 vs. T2	-1.44	0.149	-0.26
		T1 vs. T3	-2.78	**0.005**	-0.53
		T2 vs. T3	-1.39	0.165	-0.26
	Parent satisfaction	T0 vs. T1	-1.67	0.096	-0.34
		T1 vs. T2	-1.75	0.080	-0.31
		T1 vs. T3	-2.97	**0.003**	-0.56
		T2 vs. T3	-1.40	0.163	-0.26

*Note*. Significant results in bold. Significant at the *p* < 0.0125 level.

**Table 4:** Post hoc analyses for the PQRS-G scores of the intervention goals.

		*Z*	*p*	*r*
Intervention goals	T0 vs. T1	-0.07	0.944	-0.01
	T1 vs. T2	-3.13	**0.002**	-0.54
	T1 vs. T3	-2.07	0.038	-0.36
	T2 vs. T3	-1.19	0.233	-0.21

*Note*. Significant results in bold. Significant at the *p* < 0.0125 level.

## Data Availability

The data supporting the findings of this study will be made available by the authors to any qualified researcher upon reasonable request.
