# Correction to “Benefits of an Intensive Individual CO‐OP Intervention in a Group Setting for Children With DCD”

**DOI:** 10.1155/oti/9831423

**Published:** 2026-01-10

H. Krajenbrink, J. Lust, J. van Heeswijk, P. Aarts, B. Steenbergen, “Benefits of an Intensive Individual CO‐OP Intervention in a Group Setting for Children With DCD,” *Occupational Therapy International*, 2022, https://doi.org/10.1155/2022/8209128.

In the article, there is an error in Section 3.3., specifically in the descriptive results of the parent questionnaire with regard to the child′s attitude.

“As can be seen, many parents reported improvements with regard to their child′s attitude (11 out of 18), motivation (13 out of 18), and confidence (14 out of 18) in relation to motor skill activities.”

should read:

“As can be seen, many parents reported improvements with regard to their child′s attitude (9 out of 18), motivation (13 out of 18), and confidence (14 out of 18) in relation to motor skill activities.”

We apologize for this error.

